# *IL28B* genotype is associated with cirrhosis or transition to cirrhosis in treatment-naive patients with chronic HCV genotype 1 infection: the international observational Gen-C study

**DOI:** 10.1186/s40064-016-3663-6

**Published:** 2016-11-18

**Authors:** Alessandra Mangia, Victor De Ledinghen, François Bailly, Javier Brahm, Jazeps Keiss, Jonas Valantinas, Nele Rasmann, Diethelm Messinger, Fernando Tatsch, Georgios Bakalos, Graham R. Foster, on behalf of the Gen-C study group

**Affiliations:** 1 Liver Unit, IRCCS Hospital ‘Casa Sollievo della Sofferenza’, San Giovanni Rotondo, 71013 Italy; 2 Hepatology Unit, Hôpital Haut-Lévêque, CHU Bordeaux, 33600 Pessac, France; 3 Hepatology Unit, Groupe Hospitalier Nord, CHU Lyon, 69004 Lyon, France; 4 Gastroenterology Department, University of Chile Clinical Hospital, Santiago, 8380456 Chile; 5 Latvian Centre of Infectious Diseases, LLC Riga East University Hospital, Riga, 1006 Latvia; 6 Centre of Hepatology, Gastroenterology and Dietetics, Vilnius University, 08661 Vilnius, Lithuania; 7 Center for Infectious Diseases, West Tallinn Central Hospital, 10617 Tallinn, Estonia; 8 Biostatistics, PROMETRIS GmbH, 68219 Mannheim, Germany; 9 Global Medical Affairs, F. Hoffmann-La Roche Ltd, 4074 Basel, Switzerland; 10 AbbVie, North Chicago, IL USA; 11 Global Product Development Medical Affairs, F. Hoffmann-La Roche Ltd, 4074 Basel, Switzerland; 12 Institute of Cellular and Molecular Sciences, Queen Mary University of London, London, E1 2AT UK

**Keywords:** Hepatitis C, Gen-C study, IL28B, Advanced fibrosis, Chronic hepatitis C

## Abstract

**Background and purpose:**

Contradictory data exist on the association between host interleukin-28B (*IL28B*) rs12979860 genotype and liver fibrosis in patients with chronic hepatitis C (CHC). This large, international, observational study (NCT01675427/MV25600) investigated relationships between *IL28B* rs12979860 genotype and liver fibrosis stage in CHC patients.

**Methods:**

A total of 3003 adult, treatment-naive CHC patients were enrolled into the study. Patients made one study visit to provide a blood sample for genotyping; other data were obtained from medical records.

**Results:**

2916 patients comprised the analysis population; the majority were enrolled in Europe (n = 2119), were Caucasian (n = 2582) and had hepatitis C virus (HCV) genotype (G)1 infection (n = 1702) (G2 = 323, G3 = 574, G4 = 260). Distribution of *IL28B* genotypes varied according to region of enrolment, patient ethnicity and HCV genotype. A significant association was observed between increasing number of *IL28B* T alleles and the prevalence of cirrhosis/transition to cirrhosis (based on biopsy or non-invasive assessments) in G1-infected patients (CC = 22.2% [111/499], CT = 27.5% [255/928], TT = 32.3% [87/269]; p = 0.0018). The association was significant in the large subgroup of European Caucasian G1 patients (n = 1245) but not in the smaller Asian (n = 25), Latin American (n = 137) or Middle Eastern (n = 289) G1 subgroups. *IL28B* genotype was not associated with liver fibrosis stage in patients with HCV G2, G3 or G4 infection.

**Conclusion:**

This large, international study found that *IL28B* rs12979860 genotype is significantly associated with liver fibrosis stage in CHC patients with HCV G1 infection. This association was evident in European Caucasians but not in G1-infected patients from Asia, Latin America or the Middle East.

**Electronic supplementary material:**

The online version of this article (doi:10.1186/s40064-016-3663-6) contains supplementary material, which is available to authorized users.

## Background

Host interleukin-28B (*IL28B*) genotype is associated with spontaneous clearance of acute hepatitis C virus (HCV) infection, response to interferon (IFN)-based treatment, and with the development of hepatocellular carcinoma (HCC) in patients with chronic hepatitis C (CHC) (Thomas et al. 2009; Ge et al. 2009; Zhang et al. 2016). Genome-wide association studies have identified single-nucleotide polymorphisms (SNPs) in close proximity to the *IL28B* gene that encode for IFN lambda (IFN-λ), with strong associations with spontaneous or IFN-induced clearance of HCV (Thomas et al. 2009; Ge et al. 2009; Rauch et al. 2010; Suppiah et al. 2009; Tanaka et al. 2009). In particular, a host *IL28B* rs12979860 CC genotype is associated with the highest, and TT genotype the lowest, rates of response to IFN-based therapy (Thomas et al. 2009; Ge et al. 2009; Mangia et al. 2010; Asselah et al. 2012; De Nicola et al. 2012; Poordad et al. 2012; Lawitz et al. 2013; Susser et al. 2014). The T allele also increases the risk of HCC associated with HCV (Zhang et al. 2016). In addition, differences in the distribution of *IL28B* genotypes explain, in part, historical observations of low sustained virological response (SVR) rates in Black or Latino patients and high SVR rates in Asian patients (Muir et al. 2004; Yu et al. 2008; Rodriguez-Torres et al. 2009).

Despite the established role of host *IL28B* genotype as a predictor of response to therapy, the association between *IL28B* polymorphisms and the natural history of HCV is currently the subject of debate. CHC patients with an *IL28B* CC genotype had higher alanine transaminase (ALT) levels and greater hepatic necro-inflammatory activity at baseline, with worse clinical outcomes over 4 years of follow-up than patients with non-CC genotypes (Noureddin et al. 2013). However, the same study observed no difference in the rate of fibrosis progression between patients with CC and non-CC genotypes. Other studies have yielded contradictory results on the association between *IL28B* genotype and fibrosis progression (Asselah et al. 2012; Falleti et al. 2011; Fabris et al. 2011; Marabita et al. 2011; Di Marco et al. 2012; D’Ambrosio et al. 2014).

While the efficacy of recently introduced, IFN-free treatment regimens is not affected by host *IL28B* genotype, their use is limited to patients with advanced fibrosis in many jurisdictions (Lawitz et al. 2013; Jacobson et al. 2013). Therefore, predictors of fibrosis progression may provide a valuable tool for selection of patients eligible for treatment. In addition, dual peginterferon alfa/ribavirin therapy and protease inhibitor-based triple therapy continue to be used in many regions (Pawlotsky 2014). Given that advanced fibrosis is associated with lower rates of SVR to IFN-based therapies, the identification factors predictive of fibrosis stage has implications for treatment selection and optimization (Poordad et al. 2012; Lawitz et al. 2013; Hadziyannis et al. 2004; Bonnet et al. 2014; Manns et al. 2014; Jacobson et al. 2014).

The primary objective of the Gen-C study was to investigate associations between *IL28B* genotype and fibrosis stage in CHC patients, in addition to gathering further information on the distribution of *IL28B* genotypes by HCV genotype, geographic region and ethnicity. The final results from treatment-naive patients are reported here.

## Methods

### Patients and study design

Gen-C is a large, international, observational study of adults with CHC (clinicaltrials.gov, trial identifier NCT01675427). Patients were excluded if they had hepatitis B virus co-infection, a history of decompensated liver disease, major organ transplantation or end-stage renal disease. Only treatment-naive patients were included in the present analysis. Enrolment was at the discretion of the investigator.

The primary objective of the Gen-C study was to investigate the relationship between *IL28B* genotypes and liver fibrosis stage in patients with CHC.

Secondary objectives included relationships between *IL28B* genotypes and liver inflammation, ALT levels and patient demographics, and the distribution of *IL28B* genotypes by HCV genotype, geographic region and ethnicity.

### Data collection

Patients who provided written, informed consent made one study visit for blood sample collection, which was analysed in a central laboratory in Germany or Italy.

A 5 ml sample of whole blood was collected in a tube containing ethylenediaminetetraacetic acid. After collection, the tube was gently inverted 10 times and was not to be centrifuged. A bar-coded label was then applied to the tube and the sample was stored at −20 °C before being packed in dry ice and shipped by courier to the central laboratory. Samples were stored at −20 °C until DNA extraction then were thawed overnight (i.e., for approximately 16 h) and rolled for approximately 30 min before opening.

Standard polymerase chain reaction technology was used to genotype SNPs near *IL28B* (rs12979860 [CC, TC and TT] and rs8099917 [TT, GT or GG]) and inosine triphosphate pyrophosphatase (*ITPA*) (rs1127354 [AA, CA or CC] and rs7270101 [CC, AC or AA]). Positive controls for *IL28B* rs12979860 TC and rs8099917 TG were obtained from TIB MOLBIOL GmbH, Berlin, Germany. Nucleic acid purification was performed using the MagNa Pure 96 System (Roche Diagnostics, Mannheim, Germany). Genomic DNA was extracted from a 50 µL sample by adding MagNa Pure96 to make up an eluate of 100 µL. Genotype was then determined by commercial genotyping assays (LightMix Kit rs12979860 IL28B, LightSNiP rs8099917 IL28B, LightSNiP rs7270101 ITPA, LightSNiP rs1127354 ITPA; Roche Diagnostics, GmbH) using an eluate of 5 µL.

The results of previous invasive or non-invasive fibrosis assessments were entered in the electronic Case Report Form. Investigators recorded the date, type (invasive or non-invasive) and result of the assessment. Fibrosis stage was documented categorically (Cirrhosis, Transition to cirrhosis, Advanced fibrosis-non-cirrhotic, Mild/minimal fibrosis, No fibrosis). Cirrhosis/transition to cirrhosis was defined by biopsy (Ishak 4–6, METAVIR 3–4, Batts and Ludwig 3–4, Knodell 3–4 or Scheuer 3–4) or non-invasive assessment.

### Statistical analyses

Enrolment of 1500 treatment-naive and 1500 treatment-experienced patients with evaluable data was estimated to provide 80% power for a Chi square test to detect an association between liver fibrosis stage (five degrees of freedom) and *IL28B* genotype (three degrees of freedom) at a significance level of 0.05. The final enrolment target of 4000–6000 patients was arrived at after allowing for patient dropout and patients without evaluable data.

The analysis population was defined as all patients with blood samples and available data for *IL28B* SNPs. For analysis of the primary endpoint, patients were categorized as having cirrhosis/transition to cirrhosis or no cirrhosis. The relationship between cirrhosis status (cirrhosis/transition to cirrhosis vs no cirrhosis) and number of rs12979860 *IL28B* T alleles was investigated by Cochran-Armitage trend test. The relationship between *IL28B* genotype and all other fibrosis measurements (e.g. METAVIR fibrosis stage) was investigated using the Jonckheere-Terpstra trend test. The same methods were used to investigate the relationship between *IL28B* and other continuous, ordinal or binary variables, while the Pearson Chi square test was used to compare the association between *IL28B* genotypes and nominal variables.

Relationships between baseline characteristics and cirrhosis status were explored by multivariate logistic regression (MLR) analysis. Variables considered in the analysis were: alcohol consumption, ALT ratio, aspartate transaminase (AST) ratio, age, autoimmune disease, body mass index (BMI), body weight, ethnic origin, glucose intolerance/diabetes, HCV genotype, HCV RNA level, HIV–HCV co-infection, liver disease other than CHC, platelets, region of enrolment and years since HCV infection.

## Results

**Fig. 1:**
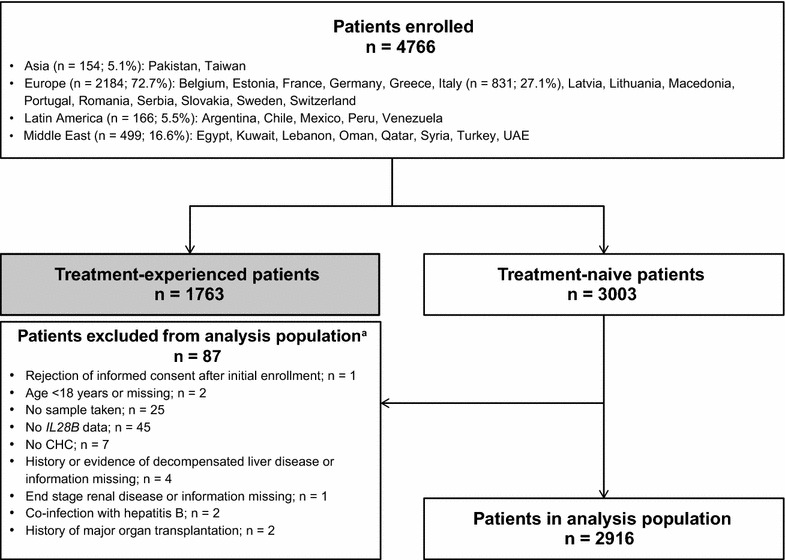
Patient flow diagram. ^a^Patients may have been excluded for more than one reason

A total of 3003 treatment-naive patients were enrolled at 213 centres across 30 countries between August 2011 (first patient) and September 2013 (last patient last visit) (Fig. 1). Overall, 87 patients were excluded, and therefore the analysis population comprised 2916 patients.

**Fig. 2:**
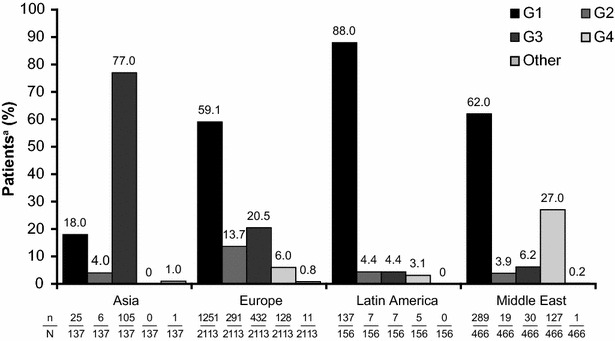
Distribution of *IL28B* rs12979860 genotype by HCV genotype and geographic region. The distribution of HCV genotype showed significant variation (p < 0.0001) by region of enrolment. p < 0.0001^b^ for association between HCV genotype and region of enrolment. ^a^Patients with multiple genotypes were assigned to disjoint groups according to the following hierarchy: G1, G4, G3, G2, other ^b^Pearson’s Chi square test

Among the total population of 2916 patients, a fibrosis assessment was conducted in 2902 individuals (99.5%), of whom 26.1% were diagnosed with cirrhosis/transition to cirrhosis (Table 1). The majority of patients were male, Caucasian and had HCV G1 infection, with similar characteristics across *IL28B* rs12979860 genotypes (Table 1). More patients with CC or CT compared with TT genotypes had a high viral load and elevated ALT levels. The distribution of HCV genotypes varied by region (Fig. 2).

**Table 1:** Patient demographics and background disease characteristics

	*IL28B* rs12979860 genotype			
	CC (n = 978)	CT (n = 1518)	TT (n = 420)	*P* value^e^
Mean age, years ± SD	46.8 ± 13.6	47.3 ± 13.2	46.8 ± 12.6	0.6973^e^
G1 (n = 1702)	47.9 ± 13.3	48.5 ± 13.2	48.1 ± 12.2	0.6602^e^
G2 (n = 323)	54.7 ± 13.1	55.1 ± 12.6	51.1 ± 15.9	0.5182^e^
G3 (n = 574)	39.2 ± 9.8	39.6 ± 10.3	39.0 ± 9.4	0.6864^e^
G4 (n = 260)	45.5 ± 13.2	44.3 ± 10.2	46.0 ± 10.4	0.7789^e^
Male, n (%)	582 (59.5)	802 (52.8)	224 (53.3)	0.0049^f^
Ethnic origin, n (%)				<0.0001^g^
Asian	89 (9.1)	83 (5.5)	9 (2.1)	
Black	5 (0.5)	24 (1.6)	19 (4.5)	
Caucasian	860 (87.9)	1349 (88.9)	373 (88.8)	
Other^a^	24 (2.5)	62 (4.1)	19 (4.5)	
Region, n (%)				<0.0001^g^
Asia	78 (8.0)	66 (4.3)	8 (1.9)	
Europe	721 (73.7)	1095 (72.1)	303 (72.1)	
Middle East	138 (14.1)	272 (17.9)	75 (17.9)	
Latin America	41 (4.2)	85 (5.6)	34 (8.1)	
BMI, kg/m^2^, mean ± SD	25.8 ± 4.4	26.0 ± 4.5	26.0 ± 4.7	0.2617^e^
HCV genotype, n (%)				<0.0001^g^
Known genotype	957 (97.9)	1501 (98.9)	414 (98.6)	
G1^b^	500 (52.2)	930 (62.0)	272 (65.7)	
G2^b^	126 (13.2)	160 (10.7)	37 (8.9)	
G3^b^	232 (24.2)	277 (18.5)	65 (15.7)	
G4^b^	92 (9.6)	129 (8.6)	39 (9.4)	
G5/6^b^	7 (0.7)	5 (0.3)	1 (0.2)	
Unknown/missing	21 (2.1)	17 (1.1)	6 (1.4)	
Duration of HCV infection, mean years ± SD	14.0 ± 12.3	14.0 ± 12.6	13.0 ± 12.4	0.1860^e^
G1 (n = 1615)	14.8 ± 13.1	14.5 ± 13.0	12.7 ± 12.5	0.0536^e^
G2 (n = 303)	16.6 ± 12.9	15.9 ± 13.5	16.6 ± 15.8	0.5129^e^
G3 (n = 552)	10.9 ± 9.6	11.8 ± 10.7	11.3 ± 8.5	0.4443^e^
G4 (n = 249)	13.7 ± 10.6	13.1 ± 10.4	14.8 ± 13.8	0.9733^e^
HIV–HCV co-infection, n (%)	38 (3.9)	49 (3.2)	15 (3.6)	0.6000^f^
Autoimmune disease, n (%)	23 (2.4)	46 (3.0)	6 (1.4)	0.6433^f^
Liver disease other than CHC, n (%)	34 (3.5)	35 (2.3)	13 (3.1)	0.3728^f^
Glucose intolerance/diabetes, n (%)	73 (7.5)	125 (8.2)	33 (7.9)	0.6614^f^
Alcohol consumption, mean units/week ± SD	1.83 ± 8.7	1.10 ± 5.2	0.87 ± 3.5	0.1423^e^
Previous/current drug use/methadone or substitute therapy, n (%)	289 (29.6)	361 (23.8)	96 (22.9)	0.0013^f^
Smoking, n (%)^b^				0.0070^g^
Never	496 (50.9)	887 (58.5)	230 (54.9)	
Previous	141 (14.5)	192 (12.7)	56 (13.4)	
Current	338 (34.7)	438 (28.9)	133 (31.7)	
ITPA rs1127354, CC, n (%)	818 (83.6)	1338 (88.1)	372 (88.6)	0.0020^f^
ITPA rs7270101, AA, n (%)	793 (81.1)	1254 (82.6)	342 (81.4)	0.6563^f^
Mean ALT, IU/L ± SD	104.1 ± 103.5	83.4 ± 79.2	72.9 ± 63.8	<0.0001^e^
Mean ALT ratio ± SD^c^	2.28 ± 2.07	1.88 ± 1.55	1.65 ± 1.31	<0.0001^e^
G1 (n = 1671)	2.09 ± 1.94	1.77 ± 1.38	1.63 ± 1.30	0.0007^e^
G2 (n = 312)	2.42 ± 2.58	1.96 ± 1.96	1.21 ± 0.829	0.0061^e^
G3 (n = 565)	2.80 ± 2.15	2.32 ± 1.91	2.05 ± 1.62	<0.0001^e^
G4 (n = 247)	2.00 ± 1.75	1.66 ± 1.11	1.65 ± 1.09	0.3885^e^
Mean HCV RNA, log_10_ IU/mL ± SD	5.94 ± 0.99	5.83 ± 0.81	5.68 ± 0.83	<0.0001^e^
HCV RNA > 800,000 IU/mL, n (%)^bd^	607/959 (63.3)	764/1498 (51.0)	179/416 (43.0)	<0.0001^f^
G1	320/495 (64.6)	493/921 (53.5)	116/268 (43.3)	<0.0001^f^
G2	81/124 (65.3)	80/157 (51.0)	23/37 (62.2)	0.1853
G3	152/231 (65.8)	131/276 (47.5)	22/65 (33.8)	<0.0001
G4	44/92 (47.8)	53/128 (41.4)	14/39 (35.9)	0.1792
Assessment of cirrhosis, n (%)				0.0723^g^
Biopsy	295 (30.2)	419 (27.6)	114 (27.1)	
Noninvasive	646 (66.0)	1046 (68.9)	289 (68.8)	
Both	37 (3.8)	46 (3.0)	12 (2.9)	
None	1 (0.1)	7 (0.5)	5 (1.2)	
Fibrosis stage (biopsy or noninvasive), n (%)				
Assessment made	976 (99.8)	1511 (99.5)	415 (98.8)	
Cirrhosis/transition to cirrhosis^b^	248 (25.4)	390 (25.8)	119 (28.7)	0.2702^f^
No cirrhosis^b^	728 (74.6)	1121 (74.2)	296 (71.3)	
Missing	2 (0.2)	7 (0.5)	5 (1.2)	

*ALT* alanine transaminase, *IL28B* interleukin-28B, *ITPA* inosine triphosphate pyrophosphatase

^a^Ethnic origin was selected from a drop-down menu in the CSR; “Other” may include patients who are Hispanic, Latino, or identified themselves as Mixed Race

^b^Percentages calculated for patients with available data only

^c^ALT ratio is ALT divided by upper limit of normal range

^d^Includes HCV RNA categories reported as ≤800,000 or >800,000 IU/mL

^e^Jonckheere-Terpstra Test for a trend of a continuous variable across the three IL28B genotype categories

^f^Cochran-Armitage Trend Test for a trend in binomial proportions across the three IL28B genotype categories

^g^Pearson Chi square test

### *IL28B* genotype and liver fibrosis stage

**Fig. 3:**
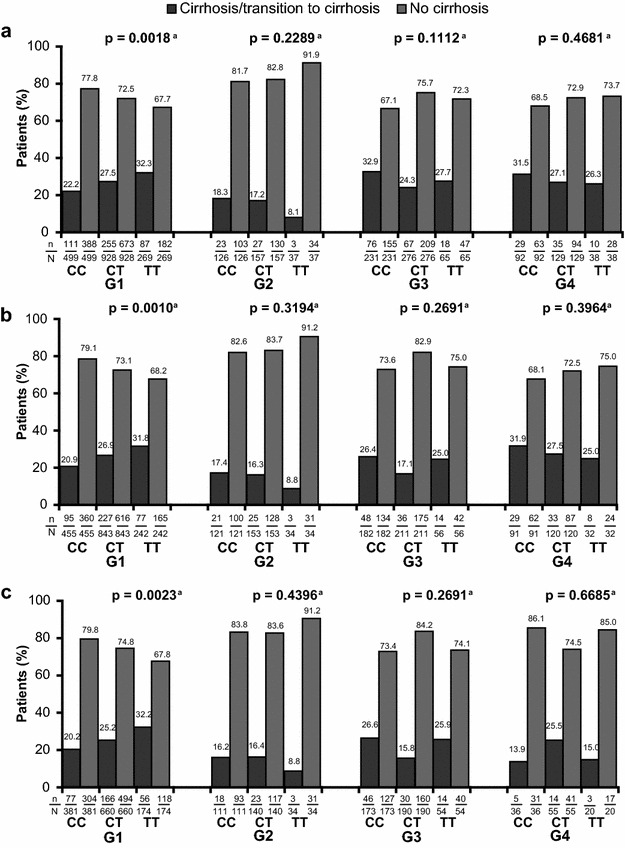
The association between *IL28B* rs12979860 genotype and liver fibrosis by HCV genotype overall (**a**), in Caucasian patients with HCV G1 infection (**b**), and in European Caucasian patients with HCV G1 infection (**c**). Significant associations were found between *IL28B* rs12979860 genotype and liver fibrosis in patients with HCV G1 infection (p = 0.0018) (**a**), in Caucasian treatment-naive patients with HCV G1 infection (p = 0.0010) (**b**), and in treatment-naive European Caucasian patients with HCV G1 infection (p = 0.0023) (**c**). ^a^Cochran-Armitage Trend Test for a trend in binomial proportions across the three IL28B genotypes

Among HCV G1-infected patients, the prevalence of cirrhosis/transition to cirrhosis increased with the number of rs12979860 T alleles overall (p = 0.0018) (Fig. 3a), in Caucasians (p = 0.0010) (Fig. 3b) and in European Caucasians (p = 0.0023) (Fig. 3c).

No statistically significant associations were observed for other HCV genotypes. Among HCV G1 patients enrolled at European study sites (n = 1245), an association was observed between the prevalence of cirrhosis/transition to cirrhosis and the number of rs12979860 T alleles (p = 0.0030, Table 2). For patients enrolled in Asia, Latin America or the Middle East, no significant associations were observed (Table 2; Additional file 1: Tables S1–S4). However, these results should be interpreted with caution due to low patient numbers.

**Table 2:** Association between liver fibrosis and *IL28B* rs12979860 genotype in patients with HCV G1 infection, by region of enrolment

Patients, n (%)	*IL28B* rs12979860 genotype			
	CC	TC	TT	p value^a^
Asia (n = 25)				
Cirrhosis/transition to cirrhosis	8 (50.0)	5 (55.6)	ND	
No cirrhosis	8 (50.0)	4 (52.3)	ND	0.7896
European (n = 1245)				
Cirrhosis/transition to cirrhosis	78 (20.2)	168 (24.8)	57 (31.7)	
No cirrhosis	309 (79.8)	510 (75.2)	123 (68.3)	0.0030
Latin America (n = 137)				
Cirrhosis/transition to cirrhosis	9 (29.0)	25 (33.8)	10 (31.3)	
No cirrhosis	22 (71.0)	49 (66.2)	22 (68.8)	0.8547
Middle East (n = 289)				
Cirrhosis/transition to cirrhosis	16 (24.6)	57 (34.1)	20 (35.1)	
No cirrhosis	49 (75.4)	110 (65.9)	37 (64.9)	0.2022

*ND* no data available

^a^Cochran-Armitage trend test for a trend in binomial proportions across the three IL28B genotype categories

### MLR analyses

**Fig. 4:**
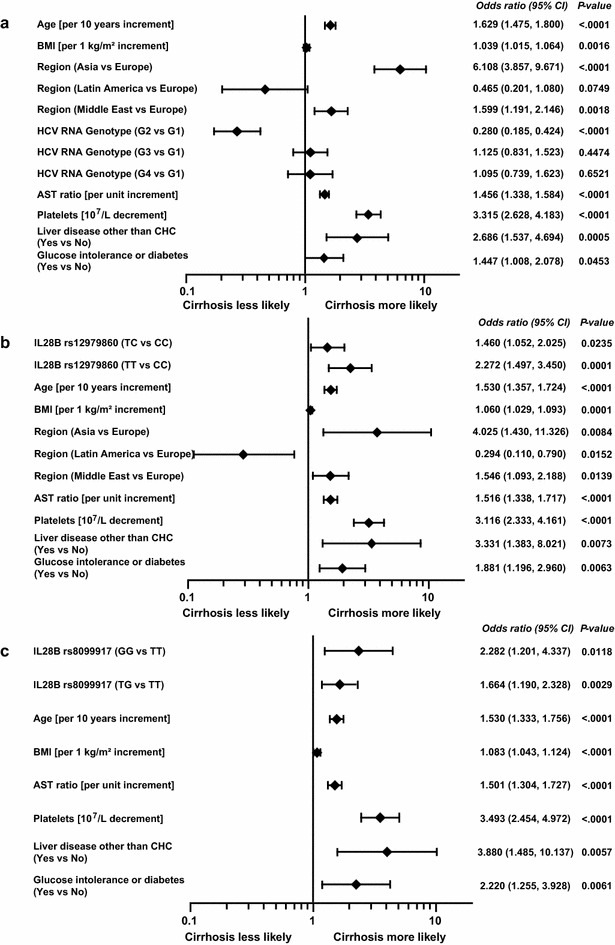
Multiple logistic regression analysis for cirrhosis or transition to cirrhosis in all patients (**a**), all patients with HCV G1 infection (**b**), and all European Caucasian patients with HCV G1 infection (**c**). *AST* aspartate transaminase; *MLR* multiple logistic regression

Older age, higher BMI, HCV G1 infection (vs G2), higher AST ratio, lower platelet count, enrolment at an Asian/Middle Eastern site (vs European), liver disease other than CHC and glucose intolerance/diabetes were significantly associated with an increased risk of cirrhosis/transition to cirrhosis (Fig. 4a). *IL28B* genotype was not associated with cirrhosis/transition to cirrhosis in the final MLR model. In contrast, when only HCV G1-infected patients were considered, rs12979860 genotype (CT or TT vs CC) was significantly associated with cirrhosis/transition to cirrhosis (Fig. 4b). When the analysis was further restricted to European HCV G1 patients, rs8099917 GG and TG genotype (vs TT) was significantly associated with cirrhosis/transition to cirrhosis (Fig. 4c).

### *IL28B* rs12979860 genotype, serum ALT levels and necro-inflammatory grade

A statistically significant decrease in mean ALT ratio was observed with an increasing number of rs12979860 T alleles for G1 (p = 0.0007), G2 (p = 0.0061) and G3 (p < 0.0001) but not G4 patients (Additional file 1: Table S5).

No association was observed between *IL28B* genotype and METAVIR fibrosis stage or necro-inflammatory grade (Additional file 1: Tables S6 and S7), or liver stiffness (Additional file 1: Table S8) overall or when stratified by HCV genotype. There was however significant association between *IL28B* genotype and AST to platelet ratio index (APRI) score and Fibrosis-4 (FIB-4) score (age, AST, platelet count and ALT) in the overall population and in the subgroup of patients with G2 and G3 infection (Additional file 1: Tables S9 and S10).

### rs12979860 genotype distributions

**Fig. 5:**
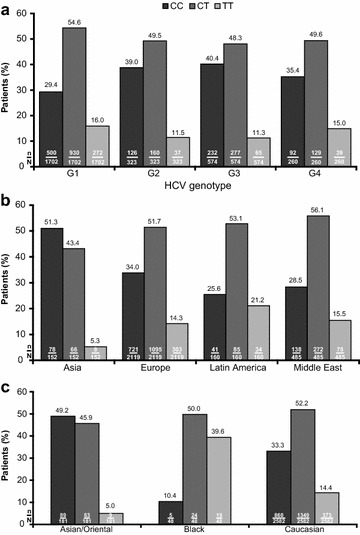
Distribution of *IL28B* rs12979860 genotype according to HCV genotype. The distribution of *IL28B* rs12979860 genotype varied significantly by HCV genotype (**a**), region of enrolment (**b**), and ethnicity (**c**). p < 0.0001^a^ for the association between HCV genotype and *IL28B* rs12979860 genotype, p < 0.0001^a^ for the association between geographic region and *IL28B* rs12979860 genotype, and p < 0.0001^a^ for the association between ethnicity and *IL28B* rs12979860 genotype. ^a^Pearson’s Chi square test

In general, an rs12979860 CT genotype was more common than a CC or TT genotype (Fig. 5a) and there was a significant association between HCV genotype and *IL28B* genotype (p < 0.0001), with the CC genotype occurring more frequently in G2 and G3 patients than in G1 and G4 patients.

rs12979860 genotype was significantly associated with region of enrolment overall (p < 0.0001, Fig. 5b) and in HCV G1 and G4 patients [p < 0.0001 and p = 0.0467, respectively (Table 3)]. rs12979860 distribution was not, however, homogenous across the countries within each region (Additional file 1: Table S8).

**Table 3:** Distribution of *IL28B* rs12979860 genotype by region of enrolment and HCV RNA genotype

HCV genotype	*IL28B* rs12979860 genotype			p value^a^
	CC	CT	TT	
G1 (n = 1702)				
Asia (n = 25)	16 (64.0)	9 (36.0)	0 (0.0)	
Europe (n = 1251)	388 (31.0)	680 (54.4)	183 (14.6)	
Middle East (n = 289)	65 (22.5)	167 (57.8)	57 (19.7)	
Latin America (n = 137)	31 (22.6)	74 (54.0)	32 (23.4)	
Total (n = 1702)	500 (29.4)	930 (54.6)	272 (16.0)	<0.0001
G2 (n = 323)				
Asia (n = 6)	4 (66.7)	2 (33.3)	0 (0.0)	
Europe (n = 291)	111 (38.1)	144 (49.5)	36 (12.4)	
Middle East (n = 19)	7 (36.8)	12 (63.2)	0 (0.0)	
Latin America (n = 7)	4 (57.1)	2 (28.6)	1 (14.3)	
Total (n = 323)	126 (39.0)	160 (49.5)	37 (11.5)	0.3572
G3 (n = 574)				
Asia (n = 105)	45 (42.9)	52 (49.5)	8 (7.6)	
Europe (n = 432)	177 (41.0)	200 (46.3)	55 (12.7)	
Middle East (n = 30)	8 (26.7)	20 (66.7)	2 (6.7)	
Latin America (n = 7)	2 (28.6)	5 (71.4)	0 (0.0)	
Total (n = 574)	232 (40.4)	277 (48.3)	65 (11.3)	0.2041
G4 (n = 260)				
Asia (n = 0)	0	0	0	
Europe (n = 128)	37 (28.9)	64 (50.0)	27 (21.1)	
Middle East (n = 127)	53 (41.7)	62 (48.8)	12 (9.4)	
Latin America (n = 5)	2 (40.0)	3 (60.0)	0 (0.0)	
Total (n = 260)	92 (35.4)	129 (49.6)	39 (15.0)	0.0467

^a^Pearson’s Chi square test for differences in IL28B rs12979860 genotype by variables

rs12979860 genotype was significantly associated with patient ethnic origin (p < 0.0001); CC was the most common genotype in Asian patients, but was infrequent in Black patients (Fig. 5c).

## Discussion

The primary finding of the Gen-C study was a positive association between an increasing number of rs12979860 T alleles and the prevalence of cirrhosis/transition to cirrhosis in treatment-naïve G1 patients. While the positive association between T allele and fibrosis stage observed for HCV G1 broadly supports findings from several previous studies, it should be taken into account that these studies did not differentiate between HCV genotypes, and also included relatively small numbers of patients (Falleti et al. 2011; Fabris et al. 2011; Di Marco et al. 2012).

There is also evidence that contradicts the results of the present study: analyses in G1 patients have reported that a CC genotype is associated with a higher prevalence of cirrhosis/transition to cirrhosis (Abe et al. 2010), and that there is no relationship between *IL28B* and fibrosis stage (D’Ambrosio et al. 2014; Bochud et al. 2012). The studies by D’Ambrosio et al. and Abe et al. measured fibrosis by biopsy; however, they included relatively small numbers of patients (D’Ambrosio et al. 2014; Abe et al. 2010). While the study by Bochud et al. (2012) included a similar number (n = 919) of Caucasian G1-infected, treatment-naive patients, the median duration of HCV infection was longer (21 vs ~14 years), and more patients had HIV–HCV co-infection (5 vs 3.5%). Two further studies found no association between rs12979860 genotype and fibrosis stage (Noureddin et al. 2013; Marabita et al. 2011).

Associations between *IL28B* genotype and liver fibrosis were restricted to HCV G1 infection in the present study. When the data were analysed by geographic region, the association between *IL28B* genotype and fibrosis status remained statistically significant in the large subgroup of European Caucasian patients with G1 infection but was not statistically significant in other populations. The lack of a relationship in the smaller subgroup of Asian, Latin American and Middle Eastern patients with G1 infection may simply be due to the smaller sample size, or may be due to factors related to ethnicity that were not measured in this analysis. Interestingly there was also a significant association between *IL28B* genotype and APRI score and FIB-4 score in the overall population and in patients with G2 and G3 infection, but not in those with G1 infection. Strong conclusions cannot be drawn regarding the overall association between *IL28B* genotype and fibrosis status, as significant associations between C allele frequency and cirrhosis/transition to cirrhosis have been reported previously (Bochud et al. 2012; Rembeck et al. 2012).

The primary immune response to HCV infection is mediated predominantly through IFN-λ cytokines and may be influenced by *IL28B* genotype (Thomas et al. 2012; Watanabe et al. 2013). Patients with a “favourable” rs12979860 CC genotype are more likely to experience spontaneous viral clearance and respond to IFN-based therapy (Thomas et al. 2009; Ge et al. 2009), while those with the T allele may be at increased risk of HCV-related HCC (Zhang et al. 2016). Interestingly, there is a well-established association between this “favourable” genotype for viral clearance (CC), and increased markers of intra-hepatic inflammatory response to HCV infection (Noureddin et al. 2013; D’Ambrosio et al. 2014; Abe et al. 2010; Bochud et al. 2012; Rembeck et al. 2012). The presence of significantly higher necro-inflammatory activity and serum ALT levels in patients with CC genotypes has been interpreted as an indication of an enhanced immune response in patients with CC genotypes as compared with non-CC genotypes (Noureddin et al. 2013; D’Ambrosio et al. 2014; Rembeck et al. 2012). A more vigorous immune response would be expected to lead to more rapid fibrosis progression over time in patients who do not experience spontaneous viral clearance. However, an association with liver fibrosis stage and *IL28B* genotype is less well established (Noureddin et al. 2013; D’Ambrosio et al. 2014; Abe et al. 2010; Bochud et al. 2012; Rembeck et al. 2012). In the current study, the CC genotype was associated with higher ALT ratios and a lower prevalence of cirrhosis/transition to cirrhosis in patients with HCV G1 infection. These results support the interpretation that the “favourable” CC genotype is associated with a more vigorous antiviral immune response but not with higher incidence of fibrosis. Unfortunately, inflammatory grade was not available for many patients, which limits the strength of this conclusion.

Recently, it has been shown that different combinations of IL28B genotype and toll-like receptor-2 (TLR-2) variants are associated with different manifestations of HCV-related liver disease and lymphoproliferative diseases (De Re et al. 2016). The *IL28B* CC genotype exerts a protective effect on CHC and the development of cirrhosis and HCC, while the TLR-2 *del/del* genotype was associated with an increased risk of HCC. In combination, the presence of one or more TLR-2 *del* alleles abolished the protective effect of the CC genotype. These data suggest that IL28B and TLR-2 are functionally interconnected in HCV disease-specific phenomena.

Baseline factors predictive of cirrhosis/transition to cirrhosis are similar to those reported elsewhere (Noureddin et al. 2013; Falleti et al. 2011; Bochud et al. 2012; Rueger et al. 2015; Wright et al. 2003). It should be noted that attendance at a clinic in Asia (vs Europe) was significantly associated with cirrhosis/transition to cirrhosis in G1 patients, which seems counterintuitive given that the CC genotype was more prevalent in Asian patients. However, this result may be explained by the greater proportion of Asian than European patients with cirrhosis at baseline (~53 vs ~25%).

Overall, the Gen-C study supports previously published frequency distributions of *IL28B* genotypes. The CC genotype was less common in G1 and G4 than G2 and G3 patients, and the proportions of CC patients in each HCV genotype agreed with previous estimates (De Nicola et al. 2012; Falleti et al. 2011; Bochud et al. 2012; Mottola et al. 2015). The geographic distribution of rs12979860 genotypes also agrees with previous reports, with the highest prevalence of the C allele in Asia, and the highest prevalence of the TT genotype in Latin America (Thomas et al. 2009; Ge et al. 2009). Finally, ethnic associations with s12979860 genotypes agree with previous reports, with the lowest prevalence of the C allele in Black patients, and the highest in Asian patients (Thomas et al. 2009; Ge et al. 2009).

Limitations include the small amount of METAVIR data collected, which tempers conclusions regarding *IL28B* genotype, inflammatory grade and fibrosis stage. Rapid fibrosis progression is associated with factors other than *IL28B* genotype, such as alcohol consumption, liver disease other than CHC, HIV–HCV co-infection and glucose intolerance, which, notwithstanding our MLR analysis, could have confounded the results.

 In summary, an increasing number of rs12979860 T alleles is associated with an increased prevalence of cirrhosis/transition to cirrhosis in treatment-naïve patients with HCV G1 infection. When the G1 population was categorized geographically, this association was evident in the large subgroup of European Caucasians with HCV G1 infection but not in the smaller subgroups of G1-infected patients from Asia, Latin America or the Middle East. Thus, the presence of the T allele may be used to prioritize treatment for G1 patients who are at increased risk of progressing to cirrhosis.

## Additional file

**Additional file 1.** List of investigators, and associations between *IL28B* genotype and liver fibrosis/inflammation in different subgroups and HCV genotypes.

## Abbreviations

ALT: alanine transaminase
AST: aspartate transaminase
APRI: AST to platelet ratio
CHC: chronic hepatitis C
HCV: hepatitis C virus
G: genotype
IFN: interferon
IL28B: interleukin 28B
MLR: multiple logistic regression
SNP: single nucleotide polymorphism
SVR: sustained virological response
TLR: toll-like receptor
