# Supplementary material for: IL28B genotype is associated with cirrhosis or transition to cirrhosis in treatment-naive patients with chronic HCV genotype 1 infection: the international observational Gen-C study
Source: Springerplus. 2016 Nov 18;5(1):1990. doi: 10.1186/s40064-016-3663-6 (PMC5116020; doi:10.1186/s40064-016-3663-6)
Supplement: Supplementary file 1 — Additional file 1. List of investigators, and associations between IL28B genotype and liver fibrosis/inflammation in different subgroups and HCV genotypes. [file 40064_2016_3663_MOESM1_ESM.pdf]

IL28B Genotype is Associated with Cirrhosis or Transition to Cirrhosis in Treatment-Naive Patients with Chronic HCV Genotype 1 Infection: the International Observational Gen-C Study: Mangia *et al.*

Supplementary Information

Study Investigators

Argentina: Gonzalo Corral, Eduardo Fassio; **Belgium:** Collins Assene, Stefan Bourgeois, Jochen Decaestecker, Christophe Moreno, Luc Lasser, Hans Van Vlierberghe, Christophe George, Hendrik Reynaert, Mike Cool, Hans Orlent, Jean Delwaide, Filip Janssens, Philippe Langlet, Geert Robaeys, Chantal De Galocsy, Jean-Pierre Mulkay, Boris Bastens, Frederik Nevens, Peter Michielsen, Matthias Dietze; **Chile:** Javier Brahm, Alejandro Soza; **Egypt:** Ahmed Monis, Asem Elfert, Imam Waked, Hesham El Khayat, Ahmed Zeid, Noman Elgarem, Ashraf Omar; **Estonia:** Triin Remmel, Riina Salupere, Krista Jaago, Nele Rasmann, Jelena Šmidt; **France:** Victor De Ledinghen, Stanislas Pol, Sophie Metivier, Dominique Guyader, Laurent Alric, Philippe Mathurin, Christophe Hézode, Daniele Botta Fridlund, Ghassan Riachi, Jean-Pierre Arpurt, Christel Rat, Thierry Sapey, Michel Doffoel, Olivier Chazouilleres, Jean-Pierre Bronowicki, François Bailly, Albert Tran; **Germany:** Tobias Goeser, Jörg Schlaak, Christian Trautwein, Steffen Zopf, Klaus Schmidt, Stefan Mauss, Christoph Berg, Christoph Antoni, Christiane Cordes, Michael-Ruppert Kraus, Luca Stein, Stefan Strahl, Manfred Kuhn, Stefan Zeuzem, Peter Malfertheiner, Kerstin Stein, Micha Loebermann; **Greece:** Dimitrios Dimitroulopoulos, Georgios Papatheodoridis, Ioannis Goulis, Maria Raptopoulou-Gigi, Konstantinos Mimidis, Epameinondas Tsianos, Georgios Dalekos, Vassiliki Nikolopoulou, Ioannis Ketikoglou, Ioannis Elefsiniotis; **Italy:** Arnaldo Andreoli, Adolfo Francesco Attili, Marcello Caremani, Danilo Tacconi, Cosimo Colletta, Carmine Coppola, Raffaele Cozzolongo, Piero Almasio, Luigi Demelia, Pietro Di Cicco, Giuseppe Foti, Roberto Ganga, Ruggiero Francavilla, Anna Giannelli, Massimo Memoli, Giuseppe Montalto, Antonio Picciotto, Antonello Pietrangelo, Alessandra Mangia, Giovanni Raimondo, Giuliano Rizzardini, Elke Erne, Giorgio Soardo, Massimo Zuin, Gabriella Verucchi, Silvia Fargion, Ludovico Tallarico, Massimo Puoti, Sergio Babudieri, Luigi Elio Adinolfi, Spartaco Sani, Maurizio Russello, Giovan Giuseppe Di Costanzo, Alfredo Di Leo, Francesco Di Lorenzo, Paolo Forte, Basilio Fimiani, Giuseppe D'Adamo, Davide Drenaggi, Fausto Ancarani, Pasquale Narciso, Ubaldo Visco Comandini, Maria Grazia Rumi, Antonio Gasbarrini, Bruno Cacopardo, Gianfranco Delle Fave, Fabio Fornari, Alessandro Grasso, Vincenzo Ostilio Palmieri, Sergio Peyre, Jacopo Vecchiet, Carlo Ferrari, Alessandra Orlandini, Giuseppe Ruggiero, Aldo Marrone, Massimo Andreoni, Nicola Passariello, Salvatore D'Angelo, Giuseppe Mazzella; **Kuwait:** Haifa Askar; Latvia: Jazeps Keiss; **Lebanon:** Ala Sharara, Zaher Houmani, Raymond Sayegh, Ahmad Dohaibi, Fouad Zaarour, Iyad Issa, Antoine Abou Rached; **Lithuania:** Limas Kupcinskas, Arvydas Ambrozaitis, Aukse Mickiene, Jonas Valantinas, Zilvinas Sukys, Giedrius Simulionis, Darius Kriukas, Aidas Kausas; **Macedonia:** Viktorija Chaloska-Ivanova, Ljubomir Ivanovski; **Mexico:** Ruben Aldrete Vazquez, Jorge Luis Poo, Rocio Torres-Ibarra, Laurent Ibarra Jose Alonso, Mauricio Castillo Barradas; **Oman:** Khalid Al Naamani; **Pakistan:** Zaigham Abbas, Asghar Auranzeb Durrani, Furqaan Ahmed, Ghias-Un Nabi Tayyab, Altaf Alam, Asad Chaudhry, Anwaar Khan, Zahid Hashmi; **Peru:** Rossana Roman, Jorge Ferrandiz; **Portugal:** Jorge

Velez, Maria João Aleixo, Paula Ferreira, Aires Figueiredo, Sara Alberto, Horácio Guerreiro, Rita Ornelas, Guilherme Macedo, Leopoldo Matos, Tiago Bana, Isabel Pedroto, Kamal Mansinho; **Qatar:** Manik Sharma; **Romania:** Liana Gheorghe, Florin Caruntu, Manuela Curescu, Ioan Sporea, Cristina Cijevschi, Eugen Dumitru, Anca Trifan, Mircea Grigorescu; **Serbia:** Milotka Fabri, Petar Svorcan; **Slovakia:** Marian Oltman, Jozef Holoman, Ivan Bunganic, Dusan Krkoska, Viera Kupcova, Lubomir Skladany; **Sweden:** Anders Lannergard, Lars Wesslén, Anders Rubenson; Switzerland: Andreas Cerny, David Semela; **Syrian Arab Republic:** Nabil Antaki; **Taiwan:** Jia-Horng Kao, Wan-Long Chuang, I-Shyan Sheen, Chi-Jen Chu, Cheng-Yuan Peng, Tsung-Hui Hu; **Turkey:** Abdulkadir Dokmeci, Seyfettin Köklü, Mehmet Demir, Meral Akdogan, Yusuf Onlen, Orhan Sezgin, Yucel Ustundag, Iftihar Koksal, Yasar Nazligul, Sebnem Gursoy, Sener Barut, Hakan Leblebicioglu, Oguz Karabay, Resat Ozaras, Rahmet Guner, Omer Basar, Osman Yuksel, Sahin Coban, Hayat Kumbasar, Ulus Akarca, Hikmet Akkiz, Mehmet Demir, Gaye Usluer; **United Arab Emirates:** Elhassan Sidahmed Elhassan Yousif, Thomas Cherukara; **Venezuela:** Marlene Dominguez, Amador Guzman, Merita Senior, Nancy Escalante, Lucy Dagher

Supplementary Table 1: Association between IL28B genotype and liver fibrosis stage by HCV genotype in patients enrolled in Asia

		***IL28B* rs12979860 genotype**			
**HCV genotype**	**n (%)**	**CC**	**CT**	**TT**	**p-value[a]**
**G1 (n=25)**	Cirrhosis or transition to cirrhosis (n=13)	8 (50.0)	5 (55.6)	ND	0.7896
	No cirrhosis (n=12)	8 (50.0)	4 (44.4)	ND	
**G2 (n=6)**	Cirrhosis or transition to cirrhosis (n=2)	1 (25.0)	1 (50.0)		0.5403
	No cirrhosis (n=4)	3 (75.0)	1 (50.0)		
**G3 (n=104)**	Cirrhosis or transition to cirrhosis (n=57)	28 (62.2)	25 (49.0)	4 (50.0)	0.2362
	No cirrhosis (n=47)	17 (37.8)	26 (51.0)	4 (50.0)	
**All other (n=16)**	Cirrhosis or transition to cirrhosis (n=2)	2 (15.4)	0 (0.0)		0.4677
	No cirrhosis (n=14)	11 (84.6)	3 (100.0)		
**Total (n=151)**	Cirrhosis or transition to cirrhosis (n=74)	39 (50.0)	31 (47.7)	4 (50.0)	0.8492
	No cirrhosis (n=77)	39 (50.0)	34 (52.3)	4 (50.0)	

ND = no data
[a] Cochran-Armitage trend test for a trend in binomial proportions across the three *IL28B* genotype categories

Supplementary Table 2: Association between *IL28B* genotype and liver fibrosis stage by HCV genotype in patients enrolled in Europe

		***IL28B* rs12979860 genotype**			
**HCV genotype**	**n (%)**	**CC**	**CT**	**TT**	**p-value[a]**
**G1 (n=1245)**	Cirrhosis or transition to cirrhosis (n=303)	78 (20.2)	168 (24.8)	57 (31.7)	0.0030
	No cirrhosis (n=942)	309 (79.8)	510 (75.2)	123 (68.3)	
**G2 (n=288)**	Cirrhosis or transition to cirrhosis (n=44)	18 (16.2)	23 (16.3)	3 (8.3)	0.3833
	No cirrhosis (n=244)	93 (83.8)	118 (83.7)	33 (91.7)	
**G3 (n=431)**	Cirrhosis or transition to cirrhosis (n=93)	46 (26.1)	33 (16.5)	14 (25.5)	0.3076
	No cirrhosis (n=338)	130 (73.9)	167 (83.5)	41 (74.5)	
**G4 (n=127)**	Cirrhosis or transition to cirrhosis (n=26)	5 (13.5)	16 (25.0)	5 (19.2)	0.4786
	No cirrhosis (n=101)	32 (86.5)	48 (75.0)	21 (80.8)	
**All other (n=16)**	Cirrhosis or transition to cirrhosis (n=5)	4 (50.0)	1 (14.3)	0 (0.0)	0.1085
	No cirrhosis (n=11)	4 (50.0)	6 (85.7)	1 (100.0)	
**Total (n=2107)**	Cirrhosis or transition to cirrhosis (n=471)	151 (21.0)	241 (22.1)	79 (26.5)	0.0823
	No cirrhosis (n=1636)	568 (79.0)	849 (77.9)	219 (73.5)	

[a] Cochran-Armitage trend test for a trend in binomial proportions across the three *IL28B* genotype categories

Supplementary Table 3: Association between *IL28B* genotype and liver fibrosis stage by HCV genotype in patients enrolled in Latin America

		***IL28B* rs12979860 genotype**			
**HCV genotype**	**n (%)**	**CC**	**CT**	**TT**	**p-value[a]**
**G1 (n=137)**	Cirrhosis or transition to cirrhosis (n=44)	9 (29.0)	25 (33.8)	10 (31.3)	0.8547
	No cirrhosis (n=93)	22 (71.0)	49 (66.2)	22 (68.8)	
**G2 (n=7)**	Cirrhosis or transition to cirrhosis (n=3)	2 (50.0)	1 (50.0)	0 (0.0)	0.4539
	No cirrhosis (n=4)	2 (50.0)	1 (50.0)	1 (100.0)	
**G3 (n=7)**	Cirrhosis or transition to cirrhosis (n=4)	0 (0.0)	4 (80.0)	ND	0.0533
	No cirrhosis (n=3)	2 (100.0)	1 (20.0)	ND	
**G4 (n=5)**	Cirrhosis or transition to cirrhosis (n=1)	1 (50.0)	0 (0.0)	ND	0.1709
	No cirrhosis (n=4)	1 (50.0)	3 (100.0)	ND	
**All other (n=4)**	Cirrhosis or transition to cirrhosis (n=1)	1 (50.0)	0 (0.0)	0 (0.0)	0.2963
	No cirrhosis (n=3)	1 (50.0)	1 (100.0)	1 (100.0)	
**Total (n=160)**	Cirrhosis or transition to cirrhosis (n=53)	13 (31.7)	30 (35.3)	10 (29.4)	0.8670
	No cirrhosis (n=107)	28 (68.3)	55 (64.7)	24 (70.6)	

ND = no data

[a] Cochran-Armitage trend test for a trend in binomial proportions across the three *IL28B* genotype categories

Supplementary Table 4: Association between *IL28B* genotype and liver fibrosis stage by HCV genotype in patients enrolled in the Middle East

		IL28B rs12979860 genotype			
**HCV genotype**	**n (%)**	**CC**	**CT**	**TT**	**p-value[a]**
**G1 (n=289)**	Cirrhosis or transition to cirrhosis (n=93)	16 (24.6)	57 (34.1)	20 (35.1)	0.2022
	No cirrhosis (n=196)	49 (75.4)	110 (65.9)	37 (64.9)	
**G2 (n=19)**	Cirrhosis or transition to cirrhosis (n=4)	2 (28.6)	2 (16.7)	ND	0.5392
	No cirrhosis (n=15)	5 (71.4)	10 (83.3)	ND	
**G3 (n=30)**	Cirrhosis or transition to cirrhosis (n=7)	2 (25.0)	5 (25.0)	0 (0.0)	0.6325
	No cirrhosis (n=23)	6 (75.0)	15 (75.0)	2 (100.0)	
**G4 (n=127)**	Cirrhosis or transition to cirrhosis (n=47)	23 (43.4)	19 (30.6)	5 (41.7)	0.4158
	No cirrhosis (n=80)	30 (56.6)	43 (69.4)	7 (58.3)	
**All other (n=19)**	Cirrhosis or transition to cirrhosis (n=8)	2 (40.0)	5 (50.0)	1 (25.0)	0.6950
	No cirrhosis (n=11)	3 (60.0)	5 (50.0)	3 (75.0)	
**Total (n=484)**	Cirrhosis or transition to cirrhosis (n=159)	45 (32.6)	88 (32.5)	26 (34.7)	0.8008
	No cirrhosis (n=325)	93 (67.4)	183 (67.5)	49 (65.3)	

ND = no data

[a] Cochran-Armitage trend test for a trend in binomial proportions across the three *IL28B* genotype categories

Supplementary Table 5: Association between *IL28B* genotype and ALT ratio

	***IL28B* rs12979860 genotype**	**n**	**Mean**	**SD**	**p-value[a]**
**Genotype 1**	CC	488	2.09	1.94	
	TC	914	1.77	1.38	
	TT	269	1.63	1.30	
	**Total**	**1671**	**1.84**	**1.56**	**0.0007**
**Genotype 2**	CC	123	2.42	2.58	
	TC	152	1.96	1.96	
	TT	37	1.21	0.83	
	**Total**	**312**	**2.05**	**2.17**	**0.0061**
**Genotype 3**	CC	228	2.80	2.15	
	TC	272	2.32	1.91	
	TT	65	2.05	1.62	
	**Total**	**565**	**2.48**	**1.99**	**<0.0001**
**Genotype 4**	CC	88	2.00	1.75	
	TC	121	1.66	1.11	
	TT	38	1.65	1.09	
	**Total**	**247**	**1.78**	**1.37**	**0.3885**
**All Other**	CC	28	1.46	1.28	
	TC	21	1.72	1.30	
	TT	7	1.33	0.70	
	**Total**	**56**	**1.54**	**1.22**	**0.3597**
**Total**	CC	955	2.28	2.07	
	TC	1480	1.88	1.55	
	TT	416	1.65	1.31	
	**Total**	**2851**	**1.98**	**1.73**	**<0.0001**

[a] Jonckheere-Terpstra Test for a trend of a continuous variable across the three *IL28B* genotype categories

Supplementary Table 6: Association between *IL28B* genotype and METAVIR fibrosis stage

			IL28B rs12979860 genotype			
**HCV genotype**	**METAVIR fibrosis (staging)**	**n**	**CC**	**TC**	**TT**	**p-value[a]**
**Genotype 1**	F0	31	6 (6.3)	20 (11.3)	5 (8.9)	
	F1	112	33 (34.7)	63 (35.6)	16 (28.6)	
	F2	106	34 (35.8)	57 (32.2)	15 (26.8)	
	F3	48	16 (16.8)	23 (13.0)	9 (16.1)	
	F4	31	6 (6.3)	14 (7.9)	11 (19.6)	
	**Total**	**328**	**95 (100.0)**	**177 (100.0)**	**56 (100.0)**	**0.5348**
**Genotype 2**	F1	13	6 (40.0)	5 (45.5)	2 (100.0)	
	F2	8	6 (40.0)	2 (18.2)	0 (0.0)	
	F3	6	2 (13.3)	4 (36.4)	0 (0.0)	
	F4	1	1 (6.7)	0 (0.0)	0 (0.0)	
	**Total**	**28**	**15 (100.0)**	**11 (100.0)**	**2 (100.0)**	**0.5469**
**Genotype 3**	F0	5	0 (0.0)	3 (11.5)	2 (22.2)	
	F1	20	8 (25.8)	10 (38.5)	2 (22.2)	
	F2	17	9 (29.0)	7 (26.9)	1 (11.1)	
	F3	13	7 (22.6)	2 (7.7)	4 (44.4)	
	F4	11	7 (22.6)	4 (15.4)	0 (0.0)	
	**Total**	**66**	**31 (100.0)**	**26 (100.0)**	**9 (100.0)**	**0.0463**
**Genotype 4**	F0	9	2 (9.5)	6 (17.1)	1 (12.5)	
	F1	29	12 (57.1)	12 (34.3)	5 (62.5)	
	F2	18	4 (19.0)	13 (37.1)	1 (12.5)	
	F3	7	3 (14.3)	3 (8.6)	1 (12.5)	
	F4	1	0 (0.0)	1 (2.9)	0 (0.0)	
	**Total**	**64**	**21 (100.0)**	**35 (100.0)**	**8 (100.0)**	**0.9393**
**All Other**	F0	1	1 (25.0)	ND	0 (0.0)	
	F1	2	0 (0.0)	ND	2 (100.0)	
	F2	2	2 (50.0)	ND	0 (0.0)	
	F3	1	1 (25.0)	ND	0 (0.0)	
	**Total**	**6**	**4 (100.0)**	ND	**2 (100.0)**	**0.3404**
**Total**	F0	46	9 (5.4)	29 (11.6)	8 (10.4)	
	F1	176	59 (35.5)	90 (36.1)	27 (35.1)	
	F2	151	55 (33.1)	79 (31.7)	17 (22.1)	
	F3	75	29 (17.5)	32 (12.9)	14 (18.2)	
	F4	44	14 (8.4)	19 (7.6)	11 (14.3)	
	**Total**	**492**	**166 (100.0)**	**249 (100.0)**	**77 (100.0)**	**0.4237**

[a] Jonckheere-Terpstra Test for a trend of a continuous variable across the three *IL28B* genotype categories

Supplementary Table 7: Association between *IL28B* genotype and METAVIR liver inflammation grade

			IL28B rs12979860 genotype			
	**METAVIR Liver Inflammation Grade**	**n**	**CC**	**TC**	**TT**	**p-value[a]**
**Genotype 1**	A0	7	1 (1.3)	4 (3.0)	2 (4.3)	
	A1	116	38 (50.0)	54 (40.9)	24 (52.2)	
	A2	95	27 (35.5)	57 (43.2)	11 (23.9)	
	A3	36	10 (13.2)	17 (12.9)	9 (19.6)	
	Total	254	76 (100.0)	132 (100.0)	46 (100.0)	0.9859
**Genotype 2**	A0	1	0 (0.0)	1 (25.0)	ND	
	A1	5	4 (50.0)	1 (25.0)	ND	
	A2	6	4 (50.0)	2 (50.0)	ND	
	Total	12	8 (100.0)	4 (100.0)	ND	0.7055
**Genotype 3**	A0	2	1 (5.6)	1 (7.1)	0 (0.0)	
	A1	17	6 (33.3)	7 (50.0)	4 (80.0)	
	A2	13	7 (38.9)	5 (35.7)	1 (20.0)	
	A3	5	4 (22.2)	1 (7.1)	0 (0.0)	
	Total	37	18 (100.0)	14 (100.0)	5 (100.0)	0.0920
**Genotype 4**	A0	6	0 (0.0)	5 (14.7)	1 (12.5)	
	A1	41	13 (61.9)	22 (64.7)	6 (75.0)	
	A2	10	7 (33.3)	3 (8.8)	0 (0.0)	
	A3	6	1 (4.8)	4 (11.8)	1 (12.5)	
	Total	63	21 (100.0)	34 (100.0)	8 (100.0)	0.0781
**All Other**	A1	5	3 (75.0)	ND	2 (100.0)	
	A2	1	1 (25.0)	ND	0 (0.0)	
	Total	6	4 (100.0)	ND	2 (100.0)	0.4795
**Total**	A0	16	2 (1.6)	11 (6.0)	3 (4.9)	
	A1	184	64 (50.4)	84 (45.7)	36 (59.0)	
	A2	125	46 (36.2)	67 (36.4)	12 (19.7)	
	A3	47	15 (11.8)	22 (12.0)	10 (16.4)	
	Total	372	127 (100.0)	184 (100.0)	61 (100.0)	0.3069

ND = no data

[a] Jonckheere-Terpstra test for a trend of a continuous variable across the three *IL28B* genotype categories

Supplementary Table 8: Association between IL28B rs12979860 genotype and liver stiffness (Fibroscan values)

HCV genotype	Liver stiffness in kPa, mean; 95% CI *IL28B* rs12979860 genotype				p-value [a]
	CC	TC	TT	All	
1	9.61; 8.51, 10.7 (n=177)	10.2; 9.22, 11.1 (n=316)	12.6; 10.3, 15.0 (n=82)	10.3; 9.64, 11.0 (n=575)	0.0605
2	11.1; 7.26, 14.9 (n=40)	7.69; 6.65, 8.72 (n=55)	7.15; 4.35, 9.94 (n=15)	8.84; 7.33, 10.4 (n=110)	0.0899
3	11.7; 9.45, 14.0 (n=60)	9.30; 7.82, 10.8 (n=83)	8.99; 6.14, 11.8 (n=24)	10.1; 8.96, 11.3 (n=167)	0.0634
4	11.4; 7.54, 15.3 (n=43)	13.4; 10.4, 16.5 (n=59)	11.1; 5.85, 16.4 (n=18)	12.4; 10.2, 14.5 (n=120)	0.6165
Other	12.1; 4.65, 19.6 (n=4)	21.9; −34.4, 78.2 (n=4)	4.00 (n=1)	15.6; −1.98, 33.1 (n=9)	0.1649
All	10.5; 9.45, 11.4 (n=324)	10.2; 9.46, 11.0 (n=517)	11.2; 9.53, 12.8 (n=140)	10.4; 9.86, 11.0 (n=981)	0.9159

[a] Jonckheere-Terpstra Test for a trend of a continuous variable across the three *IL28B* genotype categories

Supplementary Table 9: Association between IL28B rs12979860 genotype and APRI score

HCV genotype	APRI score, mean; 95% CI *IL28B* rs12979860 genotype				p-value [a]
	CC	TC	TT	All	
1	0.954; 0.867, 1.04 (n=456)	0.952; 0.876, 1.03 (n=829)	0.860; 0.756, 0.963 (n=249)	0.938; 0.886, 0.989 (n=1534)	0.0993
2	1.24; 0.969, 1.50 (n=115)	0.896; 0.725, 1.07 (n=138)	0.564; 0.400, 0.729 (n=36)	0.990; 0.853, 1.13 (n=289)	0.0009
3	1.31; 1.17, 1.44 (n=210)	1.04; 0.909, 1.17 (n=243)	0.863; 0.677, 1.05 (n=61)	1.13; 1.04, 1.21 (n=514)	<0.0001
4	1.01; 0.776, 1.25 (n=69)	0.944; 0.764, 1.12 (n=112)	0.780; 0.611, 0.949 (n=31)	0.942; 0.819, 1.07 (n=212)	0.7038
Other	1.11; 0.465, 1.75 (n=19)	1.31; 0.212, 2.41 (n=15)	0.705; 0.222, 1.19 (n=7)	1.11; 0.638, 1.59 (n=41)	0.9316
All	1.08; 1.01, 1.15 (n=869)	0.965; 0.907, 1.02 (n=1337)	0.823; 0.747, 0.900 (n=384)	0.984; 0.944, 1.02 (n=2590)	<0.0001

[a] Jonckheere-Terpstra Test for a trend of a continuous variable across the three *IL28B* genotype categories

Supplementary Table 10: Association between IL28B rs12979860 genotype and FIB-4 score

HCV genotype	FIB-4 score, mean; 95% CI				p-value [a]
	IL28B rs12979860 genotype				
	CC	TC	TT	All	
1	1.62; 1.49, 1.74 (n=455)	1.74; 1.61, 1.86 (n=826)	1.66; 1.48, 1.84 (n=247)	1.69; 1.61, 1.77 (n=1528)	0.8968
2	2.10; 1.78, 2.42 (n=115)	1.73; 1.53, 1.94 (n=138)	1.37; 1.03, 1.71 (n=36)	1.83; 1.67, 2.00 (n=289)	0.0093
3	1.73; 1.52, 1.93 (n=209)	1.42; 1.25, 1.60 (n=242)	1.32; 1.02, 1.63 (n=61)	1.53; 1.41, 1.66 (n=512)	0.0006
4	1.57; 1.27, 1.86 (n=67)	1.63; 1.38, 1.89 (n=111)	1.67; 1.25, 2.09 (n=30)	1.62; 1.44, 1.79 (n=208)	0.6459
Other	2.47; 1.59, 3.36 (n=19)	2.37; 0.596, 4.15 (n=15)	1.48; 0.670, 2.30 (n=7)	2.27; 1.54, 3.00 (n=41)	0.1737
All	1.72; 1.62, 1.82 (n=865)	1.68; 1.59, 1.77 (n=1332)	1.58; 1.44, 1.71 (n=381)	1.68; 1.62, 1.74 (n=2578)	0.0225

[a] Jonckheere-Terpstra Test for a trend of a continuous variable across the three *IL28B* genotype categories
